# Adsorption of Emerging Ionizable Contaminants on Carbon Nanotubes: Advancements and Challenges

**DOI:** 10.3390/molecules21050628

**Published:** 2016-05-12

**Authors:** Xingmao Ma, Sarang Agarwal

**Affiliations:** Zachry Department of Civil Engineering, Texas A & M University, College Station, TX 77843, USA; sarangagarwal@tamu.edu

**Keywords:** carbon nanotubes, ionizable contaminant, adsorption, surface functionalization

## Abstract

The superior adsorption capacity of carbon nanotubes has been well recognized and there is a wealth of information in the literature concerning the adsorption of unionized organic pollutants on carbon nanotubes. Recently, the adsorption of emerging environmental pollutants, most of which are ionizable, has attracted increasing attention due to the heightened concerns about the accumulation of these emerging contaminants in the environment. These recent studies suggest that the adsorption of emerging ionizable contaminants on carbon nanotubes exhibit different characteristics than unionized ones. For example, a new charge-assisted intermolecular force has been proposed for ionizable compounds because some adsorption phenomenon cannot be easily explained by the conventional force theory. The adsorption of ionizable compounds also displayed much stronger dependence on solution pH and ionic strength than unionized compounds. This article aims to present a brief review on the current understanding of the adsorption of emerging ionizable contaminants to carbon nanotubes and discuss further research needs required to advance the mechanistic understanding of the interactions between ionizable contaminants and carbon nanotubes.

## 1. Introduction

Since its first successful synthesis, carbon nanotubes (CNTs) have been widely explored as an appealing material for various industrial and commercial applications. Meanwhile, researchers in the environmental science and public health area have been actively investigating the potential toxicity and health effects of CNTs. The heightened attention in the environmental implications of CNTs derive predominantly from its unique tubular structure, very large specific surface area and great adsorption capacity and affinity with environmental pollutants [1,2].

There is a wealth of information in the literature with regard to the interactions of CNTs and environmental pollutants, especially unionized organic contaminants [3,4]. In general, CNTs possess very large adsorption capacity and strong affinity to a wide variety of organic pollutants and the process is affected by the physicochemical characteristics of both the adsorbent (CNTs) and the adsorbate (environmental pollutants). It has been shown that the nature of the CNTs, such as single-walled carbon nanotubes (SWCNTs) *vs.* multi-walled carbon nanotubes (MWCNTs) [5,6,7,8], the size and curvature of the CNTs [9], presence of defects and amorphous carbon [10,11,12], as well as the surface chemistry of CNTs [13,14,15], all play important roles in the adsorption process. For the adsorbates, the molecular structure (aliphatic *vs.* aromatic), molecular volume and presence of different functional groups strongly affect their interactions with CNTs [16,17,18]. As an example, the affinity of unionized organic compounds with CNTs typically increase from aliphatics to nonpolar aromatics to polar aromatics, due to increasingly stronger interactive forces (e.g., from Van der Waals forces to electron donor-acceptor (EDA) interactions) [5].

The interactions of CNTs and organic compounds are also strongly affected by solution chemistry, such as the ionic strength, pH and the presence of natural organic matters (NOMs) [19,20,21,22,23,24]. pH change in solution could trigger the protonation-deprotonation transition of the functional groups on CNT surface and the ionizable contaminants and cause a series of consequences such as the formation of water clusters on CNT surface [25], while ionic strength of the solution will affect the adsorption process through charge neutralization [26] and cation bridging effects [27]. Many emerging contaminants are weak acids or bases and their chemical speciation (e.g., weak acid *vs.* ionized conjugate base or weak base *vs.* ionized conjugated acid) depends on their acid dissociation constant (Ka) and solution pH. New evidences suggest that new intermolecular forces may be involved in the adsorption of ionized compounds onto CNTs due to charge assisted molecular forces (e.g., charge assisted hydrogen bonding) [28]. Natural organic matters (NOMs) and other co-existing pollutants also have a strong effect on adsorption process because of their competition for the adsorption sites [29,30,31]. The case of NOM or surfactant is even more complex due to their own strong affinity to both CNT surface and organic contaminants. On one hand, they compete with organic pollutants for adsorption sites [32], on the other hand, the NOMs could modify CNTs surface, making them more dispersed and stable and frequently increase the available surface area for adsorption [33]. These insights obtained with the study of unionized organic compounds are enlightening for the understanding of the adsorption of ionizable compounds to CNTs, yet literature has also demonstrated unique adsorption characteristics of ionizable compounds, as mentioned above. In addition, the effects of solution chemistry (e.g., pH) on the adsorption of ionizable contaminants is significantly greater than unionized organic compounds. This review aims to briefly summarize the current understanding of the adsorption of emerging ionizable contaminants on CNT surfaces and identify knowledge gaps which require further research. The review will start with a brief introduction of the emerging ionizable contaminants, followed by a survey of the literature on studies concerning the adsorption of ionizable contaminants on CNTs. The review will then present discussions on the environmental parameters which affect the adsorption of ionizable compounds and conclude with a discussion on some potential challenges which require further research to understand the interactions of emerging ionizable compounds with CNTs.

## 2. Emerging Ionizable Contaminants

Over the past century, technology and science have made unprecedented leaps in manufacturing organic chemicals for a variety of applications. Some applications have resulted in the release and accumulation of a group of emerging ionizable organic compounds. These compounds are predominantly used in a few concentrated fields such as in pharmaceuticals, personal care products (e.g., sunscreen, moisturizers, and shampoos), and flame retardants. Some of these emerging contaminants are also produced in water and wastewater treatment processes as disinfection by products. Concerns over the potential toxicity of these emerging contaminants have driven a great amount of research on the fate and transport of these emerging contaminants in the past several decades [34]. Take pharmaceuticals as an example, these compounds are specifically designed to be biologically active. Some pharmaceuticals are readily biodegradable (e.g., aspirin, ephedrine, and paracetamol), while a substantial amount of these pharmaceutical compounds could leave human body un-metabolized through excretion into wastewater effluent [35]. Examples of these not-readily biodegradable pharmaceuticals in the environment include amitriptyline, clofibrate, metronidazole, and tetracycline [36]. For those pharmaceuticals which are metabolized in human bodies, their metabolites themselves can be pharmacologically active and more toxic. The removal efficiency of pharmaceutical compounds thorough wastewater treatment is typically between 60%–90% in current treatment systems, leading to their accumulation in the environment [37]. Their presence in the environment has adverse effects on humans and wild lives unintentionally exposed to these chemicals. These chemicals also demonstrate strong effects on the microbial population in the environment and their presence has been linked to the increased resistance and mutation in microorganisms [38]. Consequently, it is important to understand the fate and transport of these emerging compounds in the environment. While these emerging contaminants are diverse, they share some common characteristics such as that they all have relatively large molecular sizes, contain one or more benzene rings and one or more functional groups. Because of the different functional groups in their molecular structures, these emerging compounds can dissociate in the environment, forming ionized compounds. The presence of charge in these ionized compounds can drastically alter the interactions of these compounds with surrounding surfaces such as soil particles and black carbon in the soil. Two recent publications summarized the adsorption of four antibiotics on different carbon materials including activated carbon, graphene oxide, carbon nanotubes and carbon nanotube/iron oxide composites [39,40]. With the continued introduction of CNTs to the environment and the strong interactions of CNTs with these emerging ionizable contaminants, it is expected that the adsorption of these contaminants on CNTs would play an important role in determining the fate and transport of these compounds in the environment and this review will focus solely on the adsorption of ionic compounds on CNTs.

## 3. Mechanisms of Adsorption of Ionizable Contaminants on CNTs

With the realization of the rapid increase of emerging ionizable contaminants in the environment and the vital role CNTs play in dictating their fate and transport, many studies have been conducted to understand their adsorption on CNTs. Oleszczuk *et al.* [41] investigated that adsorption of two pharmaceuticals on MWCNTs and found that both the oxytetracycline and carbamazepine adsorbed strongly to CNTs, but the adsorption of oxytetracycline was one magnitude greater than carbamazepine. Even though both compounds possess the amino functional group which can serve as an electron donor and form strong conjugation with the π-electrons on the benzene rings and then interact electrostatically with the positively charged regions on CNTs, oxytetracycline also contains multiple hydroxyl functional groups which could form new π–π electron donor-acceptor relationship with CNTs. The authors speculated that the hydrogen bonding between hydroxyl group and oxygen containing functional groups on CNT surfaces is relatively insignificant due to the low contents of O-containing functional groups on their CNTs. Zhang *et al.* [42] investigated the adsorption of the sulfamethoxazole on MWCNTs with different functional groups and evaluated the adsorption of different species (e.g., ionized *vs.* unionized) of this antibiotics at different pH. The authors found that regardless of the pH, the adsorption of unionized species always contributed the most (50%–80%) to the overall adsorption of sulfamethoxazole on MWCNTs. However, the charged species displayed significantly greater adsorption affinity to the MWCNTs than the unionized species. The adsorption of all species was affected by the functional groups on the CNT surface, with the hydroxyl-containing CNTs displayed greatest adsorption, followed by carboxyl-containing and graphitized CNTs. To further investigate the adsorption mechanisms, the authors added bisphenol-A in the solution to conduct competitive adsorption. The results showed that there was significant overlap between the adsorption sites of bisphenol-A and unionized sulfamethoxazole, suggesting that hydrophobic effect was a primary mechanism for sulfamethoxazole adsorption in unionized form. Electrostatic attractions of opposite charges between ionized compounds and CNT surfaces were ascribed as the main mechanism for the substantially greater adsorption affinity of ionized species than the unionized species. Yu *et al.* [43] investigated the adsorption of sulfonamides on CNTs and showed that the adsorption of all species decreased with increasing oxyl functional groups on CNT surfaces. The authors also calculated the adsorption coefficients of different species (cationic form due to the protonation of amino group, neutral form and anionic form due to the deprotonation of the sulfonamide group) and found that the adsorption coefficients of neutral species were significantly greater than charged species and cationic species had greater adsorption affinity than the anionic species, due to the electrostatic repulsion between the anionic species and the negatively charged CNTs used in their study. Interestingly, the authors noticed a linear correlation between sulfonamides adsorption and the comprehensive octanol-water coefficient (calculated as the sum of the octanol-water coefficient of each species times their relative fractions in the solution). The results suggested that hydrophobic effect was the primary mechanism for sulfonamides adsorption on CNTs. Many similar studies with different emerging ionizable compounds and differently functionalized CNTs are available in the literature. Those emerging ionizable contaminants, their functional groups, the property of CNTs, as well as the intermolecular forces involved in the adsorption are summarized in Table 1. As can be seen from the Table, similar intermolecular forces such as Van der Waals forces, hydrogen bonding, hydrophobic effect and electrostatic interactions have been involved in the adsorption of ionizable compounds on CNTs as unionized compounds. However, it should be noted that some phenomena reported in the literature with regard to the adsorption of ionizable contaminants on CNTs are either difficult to explain with those typically cited forces or a better explanation may be provided if new intermolecular forces are employed. For example, Lin and Xing [44] noticed that the adsorption of pyrogallol was significantly increased on a MWCNTs at pH 4.0–6.5 even though the compound’s octanol-water partitioning coefficient, an indication of hydrophobicity decreased. While the authors suspected that enhanced π-π interactions might explain the phenomenon, a new theory involving a charge-assisted hydrogen bonding has emerged. 

The new intermolecular force was first proposed in a recent study on the adsorption of three small aromatic acids (benzoic acid, phthalic acid and 2,6-dichloro-4-nitrophenol) on functionalized CNTs. In this study, the researchers noticed that at pH 7, where the acids are mostly dissociated, significant adsorption of these compounds occurred [65]. The result was surprising considering the significantly smaller hydrophobicity of the ionized species than their unionized counterparts. In addition, the authors noticed an increase of solution pH with the adsorption of the ionized species. Based on their thermodynamic analysis, the authors concluded that the increase of the hydrophobicity due to the conversion of ionized species to the free acid form on the CNT surface was inadequate to support the release of hydroxide ion in the solution thermodynamically. Therefore, the authors argued that new intermolecular forces must be involved between the ionized species and CNT surface and they proposed that this additional intermolecular force was the negative charge-assisted hydrogen bond (or (-)CAHB). In a subsequent study, the authors also indicated that in addition to the carboxylic acids, phenolic acids are also capable of forming such strong negative charge-assisted hydrogen bonding with CNT surfaces [66]. Through competitive adsorption studies, the researchers proposed that the CNT surface can be generally categorized into two regions: one contains the oxyl groups and are capable of forming charge-assisted hydrogen bond and the other does not contain these functional groups and are incapable of forming such strong intermolecular forces. In the second region, the hydrophobic effect plays a dominant role in adsorption. In a multi-solute system, a compound which is capable of forming charge-assisted hydrogen bonding has stronger competition on compounds with similar capability than a compound which are not able to form such strong bonds. These recent studies provide some new insights on the adsorption of emerging ionizable compounds on CNTs. However, it should be cautioned that both studies were carried out with smaller acids and the authors did notice that steric hindrance weakened the capability of molecules to form this charge-assisted hydrogen bond. As mentioned earlier, most of the emerging ionizable compounds have large molecular sizes. Therefore, the significance of these newly proposed intermolecular force for the adsorption of emerging ionizable compounds remain to be determined. 

## 4. Environmental Factors Affecting the Adsorption of Ionizable Compounds

Solution chemistry and CNT surface chemistry and characteristics have profound impacts on the interactions of organic compounds with CNTs as indicated by numerous previous studies. pH plays a significantly greater role in adsorption of emerging ionizable compounds than unionized compounds. For example, pH has been shown to significantly affect the adsorption of 1-naphthylamine, 1-naphthol and phenol on pristine and oxidized MWCNTs [67]. Understandably, due to the different pKa values of these weak acids (1-naphthol and phenol) or weak base (1-naphthylamine), the pH effect varied on those compounds. Increase of pH from 3–8 increased the adsorption of 1-naphthylamine and 1-naphthol, but decreased the adsorption of phenol. Interestingly, such pH effect is not only dependent upon the adsorbate, but also dependent upon the adsorbent because the pH effect was found to be more predominant in the oxidized MWCNTs than pristine MWCNTs. The results indicated that the modification of functional groups on the CNT surface contributed significantly to the observed pH effect on these three compounds. In a separate study, the adsorption of 1-napthylamine (pKa = 3.92) on humic acid coated MWCNTs was found to be unaffected by pH over a broad range of 2–11 while the adsorption of 1-naphthol (pKa = 9.34) on the MWCNTs increased from pH 2 to pH 8 and then became stable [68]. The reasons for the relatively constant adsorption of 1-napthyamine over a broad range of pH values on humic acid coated MWCNTs were not provided by the authors, but was probably due to the property changes of humic acids at different pHs, which compensated for the effect of 1-napthamine due to pH change. This latter study indicated that while it seems straightforward that pH will affect the dissociation of ionizable compounds with a weak acid or weak base functional group, the actual impact of pH on the adsorption of ionizable contaminants also depends on the properties of CNT surface. Therefore, the chemistry of adsorbate alone is inadequate to predict the adsorption of ionizable compounds on CNTs at different pH values.

The impact of humic substances on the adsorption of environmental chemicals on CNT surfaces is always an important consideration but it is often very complex because of the intimate interactions of the humic substances with both the adsorbent (CNTs) and the adsorbate. In general, the presence of humic substances reduces the adsorption of other environmental pollutants including ionizable contaminants due to the competition for adsorption sites, and the introduction of more polar functional groups which reduce the hydrophobicity of CNT surfaces. The physical blocking or molecular sieving could be another mechanism for the reduced adsorption of environmental pollutants in the presence of humic substances. For example, a recent study investigated that adsorption of sulfamethazine on CNTs with different surface properties and found that the presence of humic acids reduced the adsorption of sulfamethazine on all three types of CNTs, and higher concentration of humic acids led to greater reduction on the adsorption of sulfamethazine [44]. It should be mentioned that the reduction of adsorption by humic substances may be significantly higher for suspended CNTs than aggregated CNTs in that the reduced adsorption sites by humic substances on aggregated CNTs can be compensated by the increased surface area of CNTs due to higher dispersion caused by humic acids [69]. 

Ionic strength of the liquid solution is another factor which may affect the adsorption of ionizable contaminants on CNTs. Li *et al.* [70] reported that the adsorption of perfluorooctanoic acid had the highest adsorption capacity on CNTs at 1 mM ionic strength solution. Cho and colleagues investigated the impact of ionic strength on the adsorption of ibuprofen and triclosan on CNTs with different functional groups and found that ionic strength had limited impact on the adsorption of ibuprofen, but significantly reduced the adsorption of trichlosan [24]. The ionic strength effect could be due to either the charge screening on CNT surface or the facilitated aggregation of CNTs due to the compression of the electron double layer. The first impact was considered as the primary mechanism for the slightly increased adsorption of ibuprofen on CNTs while the second impact was deemed as the primary reason for the decreased adsorption of trichosan with increased ionic strength in solution. Similarly, Zhao *et al.* [46] showed that the increase of ionic strength reduced the adsorption of sulfamethoxazole, thiamphenicol on various CNTs but the impact was significantly less on carbamazepine adsorption. The authors attributed the phenomenon to the charge screening effect and the “salt-in” effect because at their tested pH, the carbamazepine was mostly in the unionized form while a great fractions of the former two pharmaceuticals were ionized. Because of the importance of electrostatic interactions for ionizable compounds, ionic strength could be a more prominent factor for the adsorption of emerging ionizable contaminants than unionized ones. Due to relatively limited studies, it is not clear whether the composition of the ionic strength will be an important consideration for the adsorption of emerging ionic contaminants. Overall, the primary mechanisms for the impact of ionic strength is that change in ionic strength could alter the thickness of electron double layers and alter the repulsion/attraction of ionizable contaminants and CNT surface and affect the aggregation of CNTs [3]. 

## 5. Future Research Needs

Despite the strides made in the past decade, mechanistic understanding on the interactions of emerging ionizable compounds with CNTs is still not fully achieved. Several hurdles remain in the road for a comprehensive understanding of the interactions of emerging ionizable compounds with CNTs. First of all, most of the mechanisms or intermolecular forces proposed in the previous literature were speculative based on measured adsorption isotherms. Few studies have made efforts for direct measurement or confirmation of the hypothesized mechanisms using advanced tools. There are only a few studies the authors are aware of that used advanced spectroscopic methods to confirm the molecular forces involved in the adsorption [44]. Future similar studies probably should include post characterization as an integral part of the adsorption study to provide more direct evidence on the molecular mechanisms involved in adsorption, in addition to the initial characterization of CNTs before adsorption study. Thermodynamics studies may be incorporated in this type of studies to obtain further insights on the nature of the intermolecular forces. In addition, current discussions on the intermolecular forces are general and qualitative at best, quantitative determination of the significance of different molecular forces and the association of molecular mechanisms with structural characteristics of ionizable compounds is still poorly understood. For example, while it is probably true that the functional groups on the emerging ionizable contaminants play a key role in governing their interactions with CNTs, will their molecular size and shape also play a role? These parameters are mostly excluded from the discussion in current adsorption studies, but may be potentially important for the interpretation of the differences in the adsorption of different ionizable compounds. For instance, a recent study investigated the adsorption of nalidixic acid and 2-(4-methylphenoxy)ethanol on different carbon nanotubes and found that while the adsorption affinity of nalidixic acid was bigger than that of 2-(4-methylphenoxy)ethanol on all CNTs, the adsorption capacity of the latter was higher than the former because its relatively smaller molecular size allowed it to occupy some micropores in CNT aggregates [71]. Atomic simulation may function as a significant complementary approach for experimental investigations on the adsorption of emerging ionic contaminants on CNTs. The detailed approach for atomic simulation is beyond the scope of this review, but the advantages, limitations and examples of applications have been broadly reported in the literature [72,73].

Lack of predictable models to estimate the adsorption capacity of ionizable compounds is another area which requires continued efforts on the adsorption of emerging ionizable contaminants. Currently, the adsorption coefficient of ionizable compounds is estimated by the sum of the adsorption coefficients of different species times their relative fractions in the solution. The relative fractions of different species at different pH are governed by the acid dissociation constants of the ionizable compounds and the solution pH and can be very accurately estimated. However, the adsorption coefficient of different species at different pH may vary because the protonation/deprotonation of the functional groups on CNT surfaces. Therefore, the assumption that the adsorption coefficients of different species on CNTs maintain constant over a broad pH range needs further scrutiny. In addition, due to the co-presence of unionized and ionized species, their competitive adsorption could be a significant factor affecting the adsorption of these molecules to CNTs. However, competitive studies between the unionized species of these molecules with their own conjugates are rare. One of the previous studies suggested that unionized compounds are competitive for the adsorption of ionizable compounds but not *vice versa* [45]. However, the generality of this statement is uncertain and should be further investigated because the extent of competitive adsorption may also depend on the surface properties of CNTs. Additionally, for this matter, how the co-existing chemicals, in particular, the organic matter affect the adsorption of emerging ionizable contaminants is not clear. Previous studies with other carbon-based adsorbents may provide some insights. In previous studies on the impact of natural organic matter on the adsorption of organic contaminants, limited description was provided on the characteristics of natural organic matter. Considering the complexity of the molecular structures of natural organic matters and their close interactions with both organic contaminants and the CNTs, detailed information on the natural organic matter itself is needed to obtain mechanistic insights on its role in the adsorption of ionizable compounds on CNTs. 

In closing, the knowledge on the adsorption of emerging ionizable contaminants on variously functionalized CNTs has grown rapidly in the last 5–10 years. The literature appears to suggest that new intermolecular forces might have contributed to the adsorption of ionizable compounds on CNTs, in addition to the molecular forces which have affected the adsorption of unionized compounds on CNTs. The literature clearly indicates a stronger effect of pH and ionic strength on the adsorption of ionizable compounds than unionized species. With these new general understandings, however, more mechanistic insights are needed to unravel the adsorption of ionizable compounds on CNTs. Suggestions for future research include better post adsorption characterization of CNTs to obtain direct evidences on adsorption mechanisms, and taking into consideration the detailed properties of evaluating parameters such as the composition of the ionic strength and structural and chemical properties of natural organic matter, rather than provide only a general description of these parameters.

## Figures and Tables

**Table 1:** Summary of the adsorption of emerging ionizable contaminants on carbon nanotubes (CNTs).

Compound	Functional Groups	Multi/Single-Walled Carbon Nanotubes (MWCNTs/SWCNTs)	Mechanisms	Ref.
Sulfamethazine	Amine, Methyl pyrimidine	Pristine, C-MWCNTs ^a^ H-MWCNT ^b^	π–π interactions, hydrogen bonding interactions, hydrophobic interaction, Lewis acid-base interaction	[45,46]
Cephalexin	Amino, Phenylacetamido	MWCNTs	not specified	[47]
Triclosan	Phenol, Chlorine	SWCNTs MWCNTs O-MWCNTs ^c^	electrostatic interactions, hydrophobic interactions	[24]
Ciprofloxacin	Carboxylic acid, Cycloamine	SWCNTs H-MWCNTs G-MWCNTs ^d^ C-MWCNT	hydrophobic, π-π electron-donor-acceptor (EDA) interactions electrostatic interactions	[48,49]
Aspirin	Carboxylic acid, Ester	MWCNTs	not specified	[50]
Isoproturon	Isopropyl, Carbonyl, Amine	MWCNTs	electrostatic interactions	[51]
Paracetamol	Hydroxyl, Amide	Polyaniline-MWCNTs ^e^ C-MWCNTs	not specified	[52,53]
Amitriptyline	Amine	MWCNTs	π–π selective interactions	[54]
Metronidazole	Nitro	SWCNTs MWCNTs	π–π stacking, hydrophobic and electrostatic interactions	[55,56]
Tetracycline	Phenol, Amino, Alcohol	SWCNTs MWCNTs	Van der Waals forces, π–π electron-donor-acceptor (EDA) interactions, cation-π bonding	[20]
Carbamazepine	Amide	SWCNTs MWCNTs	π–π electron-donor-acceptor (EDA) interactions, hydrogen bonding hydrophobic interaction, Lewis acid-base interaction	[41,46,57]
Metoprolol	Phenol, Amine, Ether	MWCNTs	electrostatic interactions	[58,59]
Sulfamethoxazole	Sulfamoyl, Amine	MWCNTs	π–π electron coupling	[46,60]
Diclofenac	Phenylacetic acid, Chlorine, Amine	MWCNTs	electrostatic interactions, hydrophobic interaction, hydrogen bonding, Lewis acid-base interaction	[46,51]
Sulfadimethoxine	Amine	MWCNTs	hydrophobic effect, hydrogen bonding	[61]
Bisphenol A	Phenol	SWCNTs MWCNTs C-MWCNTs	electrostatic interactions, π–π interactions, hydrophobic effect	[62,63]
Bisphenol AP	Phenol	MWCNTs	hydrogen bond, hydrophobic effect, π−π stacking interactions	[64]
Chloramphenicol	Chlorine, Nitro, Amide	MWCNTs	π–π EDA interactions, hydrophobic interaction, hydrogen bonding, Lewis acid-base interaction	[46]
Thiamphenicol	Chlorine, Sulfonyl, Amide	MWCNTs	π–π EDA interactions, hydrophobic interaction, hydrogen bonding, Lewis acid-base interaction	[46]
Florfenicol	Chlorine, Flourine, Amide	MWCNTs	π-π EDA interactions, hydrophobic interaction, hydrogen bonding, Lewis acid-base interaction	[46]

^a^: C-MWCNTs: carboxylated-MWCNTs; ^b^: H-MWCNTS: hydroxylated-MWCNTs; ^c^: O-MWCNTs: oxidized-MWCNTs; ^d^: G-MWCNTs: graphitized-MWCNTs; ^e^: P-MWCNTs: polyaniline-MWCNTs.
